# Dengue fever complicated with Guillain-Barré syndrome: a case report and review of the literature

**DOI:** 10.1186/s13256-018-1626-y

**Published:** 2018-05-15

**Authors:** Chamara Dalugama, John Shelton, Mahendra Ekanayake, Indika Bandara Gawarammana

**Affiliations:** 1 Department of Medicine, University of Peradeniya, Galaha Road, Peradeniya, Sri Lanka; 2 University Medical Unit, Teaching Hospital, Peradeniya, Sri Lanka

**Keywords:** Dengue fever, Guillan-Barré syndrome, Respiratory failure, Sri Lanka

## Abstract

**Background:**

Dengue is an arboviral infection classically presenting with fever, arthralgia, headache, and rashes. It is hyperendemic in Sri Lanka and has a major impact on health. Neurological complications of dengue fever are rare but have been reported in the literature.

**Case presentation:**

A 60-year-old Sri Lankan man presented with a history of fever, arthralgia, and generalized malaise of 2 days duration. A diagnosis of dengue was confirmed with leukopenia, thrombocytopenia, and positive NS1 antigen done on day 2 without evidence of hemoconcentration. On admission, our patient had weakness of the bilateral lower limbs, which progressed in an ascending pattern involving both upper limbs and neck muscles, requiring assisted ventilation. Electromyography confirmed a demyelinating polyneuropathy and cerebrospinal fluid showed albumincytological dissociation. He was treated with intravenous immunoglobulins and made an uneventful recovery. Subsequently, his immunoglobulin M test result for dengue virus was positive.

**Conclusions:**

Guillain-Barré syndrome is a rare but possible neurological sequel following dengue fever. In regions where dengue is hyperendemic, screening for dengue illness may be important in patients presenting with acute flaccid paralysis.

## Background

Dengue is an arboviral infection commonly presenting with fever, arthralgia, headache, and rashes. It is a major global public heath challenge. Neurological manifestations of dengue fever are rare but have been reported in the medical literature.

Guillain-Barré syndrome (GBS) is a demyelinating polyneuropathy which frequently follows gastrointestinal or respiratory infections. Although rare, few cases of GBS have been causally linked to serologically confirmed dengue illness in the medical literature. Etiopathogenesis of GBS following dengue is not yet full described. But the molecular mimicry causing immune attack on myelin and axons, and pro-inflammatory cytokines such as tumor necrosis factor (TNF), interleukins, and complements participating in immune response are postulated as possible mechanisms. Plasma exchange and intravenous immunoglobulins are equally effective and a mainstay of management.

Patients with dengue fever can develop acute flaccid paralysis as a complication. In regions where dengue is hyperendemic, screening for dengue illness may be important in patients presenting with acute flaccid paralysis.

## Case presentation

A 60-year-old Sri Lankan man was admitted in April 2017 with a 2-day history of fever with arthralgia, myalgia, headache, and generalized malaise. He complained of numbness and pain of the bilateral upper limbs and lower limbs, with weakness of both lower limbs. He was unable to walk as usual or get up from a squatting position. He could pass urine without difficulty and had no difficulty in breathing and coughing. He denied recent diarrheal, respiratory illness or recent vaccinations. He was previously apparently well with no significant comorbidities.

On examination, he was conscious, rational, and had normal vital parameters. Cardiovascular, respiratory and abdominal examinations were normal. A limb examination revealed hypotonia and reduced power in the bilateral lower limbs. His upper limbs were normal. His lower limb tendon reflexes were absent with reinforcement and his upper limb reflexes were diminished. All his sensory modalities were intact. Although he had a good cough reflex, his neck muscle power was reduced. A cranial nerve examination was normal. On admission, his spontaneous tidal volume (STV) was 400 mL. A provisional diagnosis of Guillan-Barré syndrome was made.

The complete blood count on admission showed a white cell count of 4.2 × 10^6^/microL, Platelets of 166 × 10^3^/microL and a hematocrit of 40. Hus nonstructural protein 1 (NS1) antigen result was positive on admission. With the compatible history, positive dengue antigen, leukopenia and thrombocytopenia, a diagnosis of dengue fever was made. Serology results for HIV, hepatitis B and a throat swab for influenza were negative.

Nerve conduction studies revealed grossly delayed nerve conduction in common peroneal and posterior tibia nerves. F waves were delayed. Ulnar nerve conduction was delayed with absent F waves. It was compatible with a severe demyelinating polyneuropathy. A cerebrospinal fluid study done later on day 11 of his illness showed albumincytological dissociation (protein 70 g/dL, cell count - lymphocytes 5/cumm and no polymorphs).

Our patient was started on intravenous immunoglobulins (IvIG) 0.4 g/kg/day (30 g in this 75 kg weighing man) on admission. On the second day of hospital stay, our patient deteriorated neurologically. He was having poor respiratory effort with low neck muscle power, and his spontaneous tidal volume dropped to 150 mL. He was electively paralyzed and intubated. He was ventilated for 3 days and intravenous immunoglobulins were administered for a total of 5 days. He made a remarkable recovery and was extubated on day 4 of IvIG. He was able to walk without support on discharge.

The dengue illness of our patient followed an uncomplicated course without clinical or ultrasonic evidence of hemoconcentration. Lowest thrombocytopenia noted was 32 × 10^3^/microL on the fourth day of his illness. Transaminases were marginally elevated (AST > ALT). Both dengue virus-specific immunoglobulin M (IgM) and immunoglobulin G (IgG) were positive on the sixth day of his illness. On discharge, our patient was fully recovered neurologically.

## Discussion

Dengue fever is an arboviral infection classically presenting with fever, arthralgia, headache, and rashes. Dengue virus belongs to the family flaviviridae and there are four serotypes referred to as DEN 1 to DEN 4 [1]. The World Health Organization (WHO) considers dengue as a major global public health challenge in the tropics and subtropic regions [2]. Dengue virus can cause a spectrum of disease varying from asymptomatic illness to dengue fever (DF) to the severe illness of dengue hemorrhagic fever (DHF) [3]. Dengue fever has become endemic in Sri Lanka since the 1960s and the incidence had increased dramatically over the past three decades [4].

We describe a case of Guillain-Barré syndrome (GBS) associated with a proven episode of dengue fever. In this patient, dengue fever was not severe as our patient did not develop hemoconcentration and plasma leak. But he needed ventilatory support following respiratory failure subsequent to GBS.

Neurological manifestations of dengue fever are uncommon, but reported in the medical literature. Verma *et al*. described neurological complications of patients with positive serology for dengue fever [5]. These were categorized into three groups on the basis of possible pathological mechanisms:

1. Neurotrophic complications such as encephalitis, myelitis, or myositis
2. Systemic complications such as hypokalemic periodic paralysis
3. Post-infectious immune-mediated complications such as GBS, opsclonus myoclonus syndrome

Neurological complications are reported to occur in 0.5–6% of cases with dengue fever [6].

Guillain-Barré syndrome is an acute, frequently severe, mainly a demyelinating polyradiculopathy that is autoimmune in nature [7]. Approximately 70% of cases of GBS occur 1–3 weeks following an acute infection, commonly respiratory or gastrointestinal. Cytomegalovirus, Epstein-Barr virus, *Campylobactor jejuni*, Mycoplasma are commonly identified agents [7]. GBS is an uncommon neurological sequel of dengue fever and the neurological picture induced by the dengue virus is similar to GBS caused by other infections [8].

Fragoso *et al*. described ten cases of GBS with dengue fever. Neurological manifestations in this case series were often severe, but recovery was mostly as complete and fast as in our case described here [9]. Soares *et al*. discussed about seven cases of GBS associated with positive dengue serology. In his case series, he emphasized the fact that dengue infection to be routinely looked for in GBS cases in the endemic zone [10]. In 2012, Qureshi *et al*. reported a case of both upper and lower limb weakness developed in an ascending pattern following a febrile illness of 3 days. Subsequently, the diagnosis of GBS was confirmed by nerve conduction studies and dengue IgM was positive in serum [11]. Similarly, Chew *et al*. reported two cases of post dengue GBS; the first case was a 43-year-old woman with serologically confirmed dengue fever developing acute flaccid paralysis requiring assisted ventilation and the second case of a 51-year-old man with bilateral facial nerve palsy and areflexia without motor weakness. Both made a full recovery [12]. Sharma *et al*. in 2011 reported a patient who presented with 3 days’ history of fever and weakness of all four limbs. Nerve conduction confirmed an acute motor sensory axonal-type variant of GBS with thrombocytopenia and positive dengue serology. The patient improved with IvIG and supportive therapy [13]. Patel *et al*. reported a 30-year-old man presented with bilateral facial nerve palsy and a history of fever with thrombocytopenia. Nerve conduction revealed a demyelinating polyneuropathy and serum was positive for dengue virus-specific IgM [14].

It is suggested that the clinical manifestations of GBS are the result of cell-mediated immunological response to nonself-antigens that misdirect to host nerve tissue. This is known as molecular mimicry [15]. This immune injury can be directed toward the myelin or axons of peripheral nerves. Pro-inflammatory cytokines that participate in the immune response of dengue fever may have an important role in the pathogenesis of GBS. These chemical substances such as TNF, complements, and interleukins may have a causal role in etiopathogenesis of GBS [16]. Garg *et al*. reviewed 29 patients with dengue fever associated with GBS and the majority of the patients had low platelet counts while the patient is developing the weakness, suggesting that GBS was a manifestation of acute dengue fever [17]. Similarly, our patient described in the current case report developed the weakness while having the acute infection, suggesting that pro-inflammatory cytokines in the immune response of dengue would have a more important role than molecular mimicry as a post-infectious sequel in the pathogenesis of dengue fever.

In a meta-analysis of six phase 2 trials comparing plasma exchange to supportive care alone in GBS found that patients treated with plasma exchange had significantly better outcome measures including time to recover walking without aid, percentage of patients requiring artificial ventilation, duration of ventilation, full muscle strength recovery after 1 year, and severe sequel after 1 year [18]. A randomized controlled trial on intravenous immunoglobulin (IvIG) in 1992 showed that IvIG is equally as effective as plasma exchange [19].

Our patient was treated with intravenous immunoglobulins (0.4 g/kg) for 5 days and showed a marked clinical improvement. It was preferred over plasma exchange based on its low side effect profile and ease of administration.

In Sri Lanka, where dengue is hyperendemic, patients may present to healthcare solely with unusual neurological manifestations such as GBS, myelitis or myositis, and the possibility that they might harbor dengue illness must be borne in the mind of the physician.

## Conclusions

Dengue fever is hyperendemic in Sri Lanka. It can rarely present with various neurological manifestations. GBS is an uncommon neurological sequel of dengue fever, which is not well documented in medical literature globally. Thus our case report calls attention to the possibility of GBS may occur in association with dengue fever and the need to consider the possibility of dengue fever in a hyperendemic area in patients presenting with acute flaccid paralysis.

## References

[1] Halstead SB. Pathogenesis of dengue: challenges to molecular biology. Science. 1988;239:476–81. 10.1126/science.3277268 3277268

[2] Guzman MG, Halstead SB, Artsob H, Buchy P, Farrar J, Gubler DJ, et al. Dengue: a continuing global threat. Nat Rev Microbiol. 2010;8(Suppl):S7–16. 10.1038/nrmicro2460 PMC4333201 21079655

[3] World Health Organization (WHO). Dengue. Guidelines for diagnosis, treatment, prevention and control. New ed. Geneva, Switzerland: World Health Organization; 2009. 23762963

[4] Kularatne SAM, Gawarammana IB, Kumarasiri PR. Epidemiology, clinical features, laboratory investigations and early diagnosis of dengue fever in adults: a descriptive study in Sri Lanka. Southeast Asian J Trop Med Public Health. 2005;36(3):686–92. 16124439

[5] Verma R, Sharma P, Garg RK, Atam V, Singh MK, Mehrotra HS. Neurological complications of dengue fever: experience from a tertiary center of north India. Ann Indian Acad Neurol. 2011;14(4):272–8. 10.4103/0972-2327.91946 PMC3271466 22346016

[6] Hendarto SK, Hadinegoro SR. Dengue encephalopathy. Acta Paediatr Jpn. 1992;34:350–7. 10.1111/j.1442-200x.1992.tb00971.x 1509881

[7] Hauser SL, Amato AA. Gullain-Barre syndrome and other immune-mediated neuropathies. In: Longo DL, Fauci AS, Kasper DL, Hauser SL, Jameson JL, Loscalzo J, editors. Harrison’s Principles Internal Medicine. 18th ed. New York: McGraw Hill; 2011. p. 3473–7.

[8] Esack A, Teelucksingh S, Singh N. The Guillain-Barré syndrome following dengue fever. West Indian Med J. 1999;48:36–7. 10375992

[9] Fragoso YD, Gomes S, Brooks JBB, Matta AP d C, Ruocco HH, Tauil CB, Sousa NA d C, Spessotto CV, Grippe T. Guillain-Barré syndrome and dengue fever: report on ten new cases in Brazil. Arq Neuropsiquiatr. 2016;74(12):1039–40. 10.1590/0004-282X20160161 27992005

[10] Soares CN, Cabral-Castro MJ, Peralta JM, Freitas MR, Puccioni-Sohler M. Oligosymptomatic dengue infection: a potential cause of Guillain Barre syndrome. Arq Neuropsiquiatr. 2008;66:234–7. 10.1590/s0004-282x2008000200018 18545789

[11] Qureshi NK, Begum A, Saha PR, Hossain MI. Guillain–Barre syndrome following dengue fever in adult patient. J Med. 2012;13:246–9.

[12] Chew NK, Goh KJ, Omar S, Tan CT. Guillain–Barre syndrome with antecedent dengue infection: a report of two cases. Neurol J Southeast Asia. 1998;3:85–6.

[13] Sharma CM, Kumawat BL, Ralot T, Tripathi G, Dixit S. Guillain-Barre syndrome occurring during dengue fever. J Indian Med Assoc. 2011;109(9):675–82. 22480104

[14] Patel S, Ranjan R, Verma R, Agrawal CS, Gupta P. Bilateral facial weakness following dengue fever. Neuroimmunol Neuroinflammation. 2016;3:63–4.

[15] Hughes RAC, Hadden RDM, Gregson NA, Smith KJ. Pathogenesis of Guillain Barré syndrome. J Neuroimmunol. 1999;100:74–97. 10.1016/s0165-5728(99)00195-2 10695718

[16] Shah I. Dengue presenting as Guillain–Barre syndrome. Dengue Bull. 2007;31:166–8.

[17] Garg RK, Malhotra HS, Jain A, Malhotra KP. Dengue-associated neuromuscular complications. Neurol India. 2015;63:497–516. 10.4103/0028-3886.161990 26238884

[18] Hughes RA, Wijdicks EF, Barohn R, Benson E, Cornblath DR, Hahn AF, et al. Practice parameter: immunotherapy for Guillain-Barré syndrome: report of the Quality Standards Subcommittee of the American Academy of Neurology. Neurology. 2003;61:736–40. 10.1212/wnl.61.6.736 14504313

[19] van der Meché FG, Schmitz PI. A randomized trial comparing intravenous immune globulin and plasma exchange in Guillain-Barré syndrome. Dutch Guillain-Barré Study Group. N Engl J Med. 1992;326:1123–9. 10.1056/NEJM199204233261705 1552913

